# Supplementary figures and images for: Clinical usefulness of serum autotaxin levels for predicting decompensation development and prognosis in patients with compensated cirrhosis
Source: PLoS One. 2026 Apr 9;21(4):e0347310. doi: 10.1371/journal.pone.0347310 (PMC13065023; doi:10.1371/journal.pone.0347310)

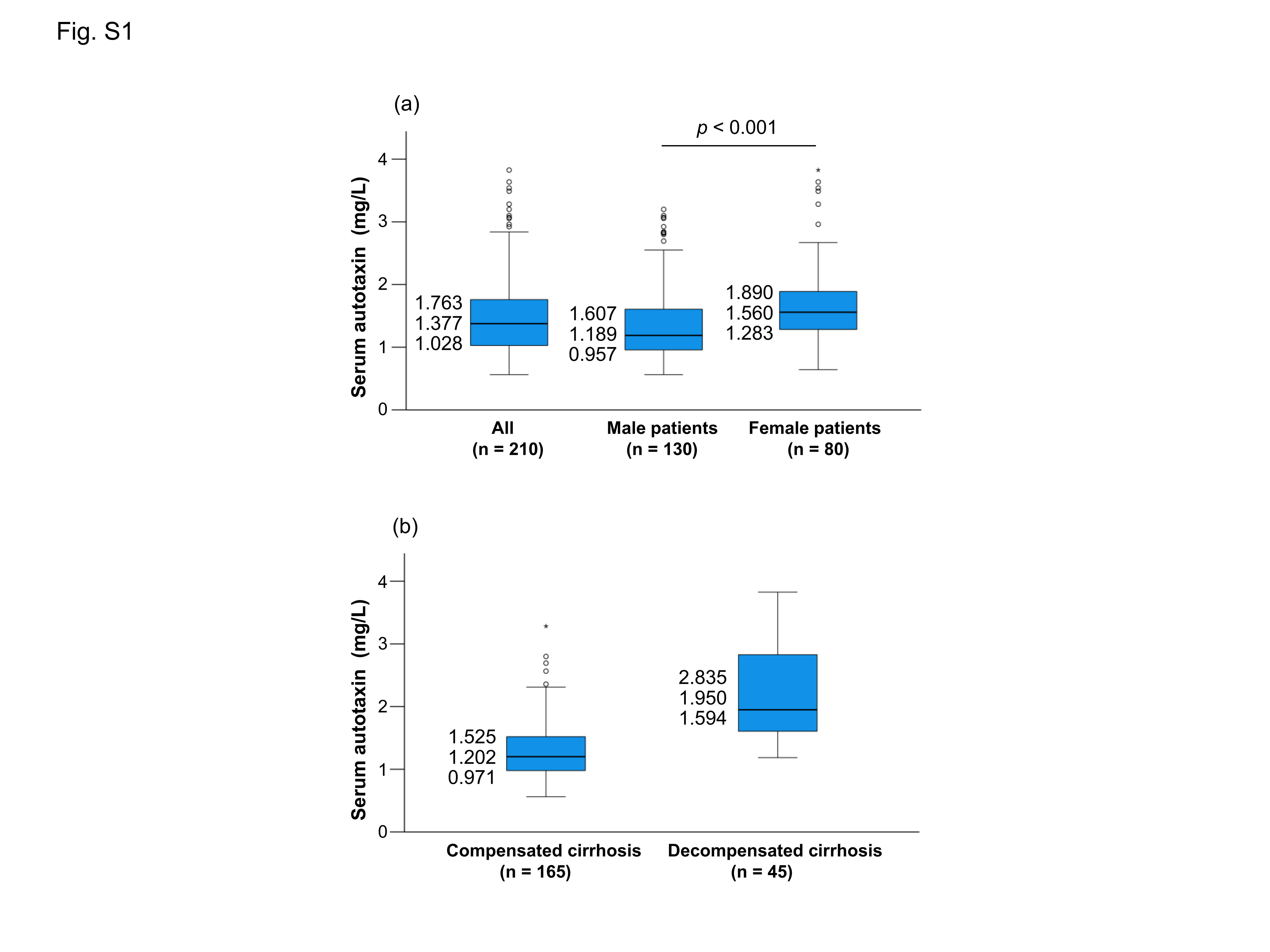

Supplement: S1 Fig — (a) Male vs. female patients; (b) Compensated vs. decompensated cirrhosis. The numbers indicate the 3rd quartile, median, and 1st quartile, respectively, from top to bottom. (TIF) [file pone.0347310.s001.TIF]

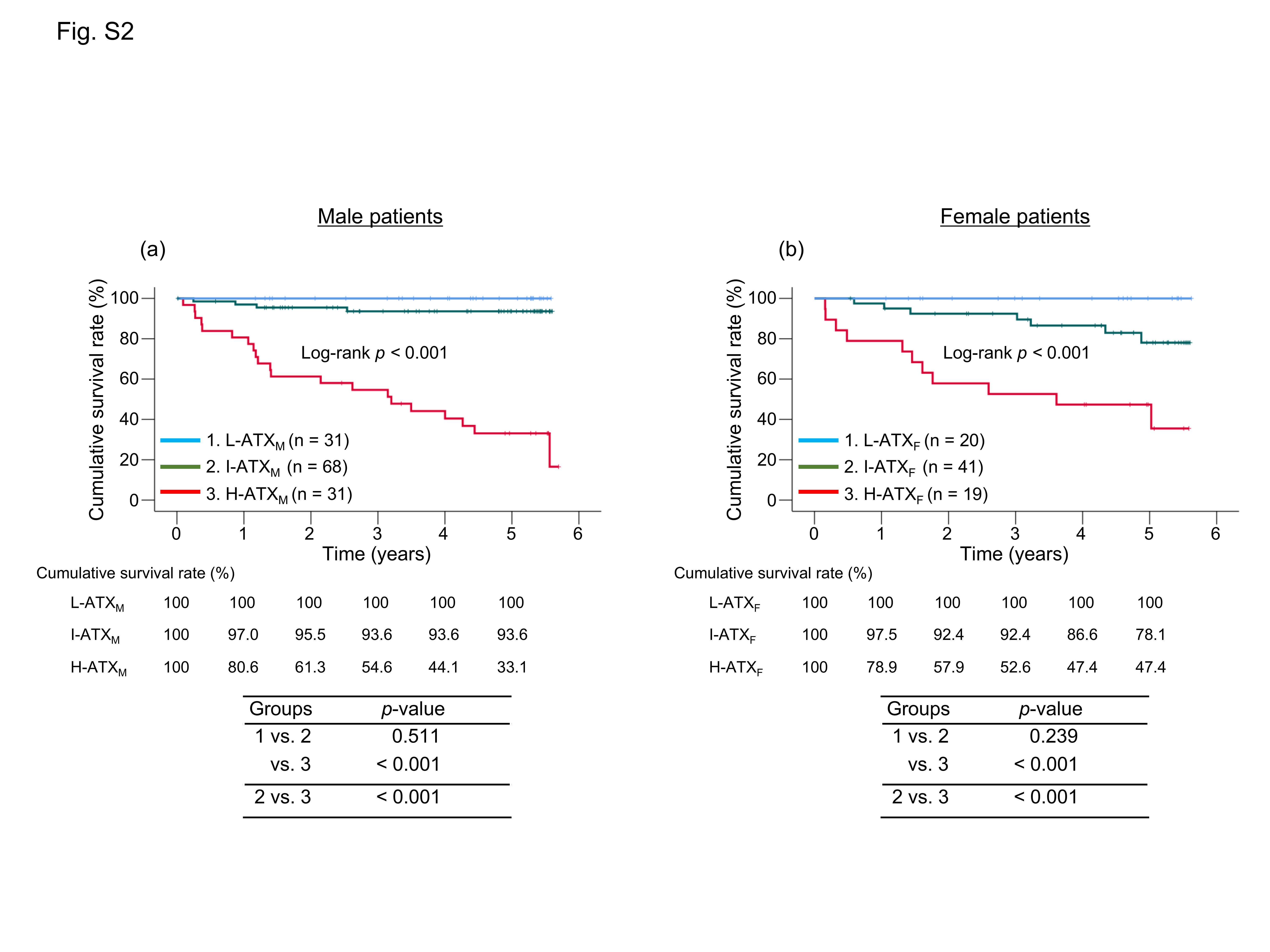

Supplement: S2 Fig — (a) Male patients; (b) female patients. L-ATXmen (M)/women (W), low-ATX group; I-ATXM/W, intermediate-ATX group; H-ATXM/W, high-ATX group. (TIF) [file pone.0347310.s002.TIF]

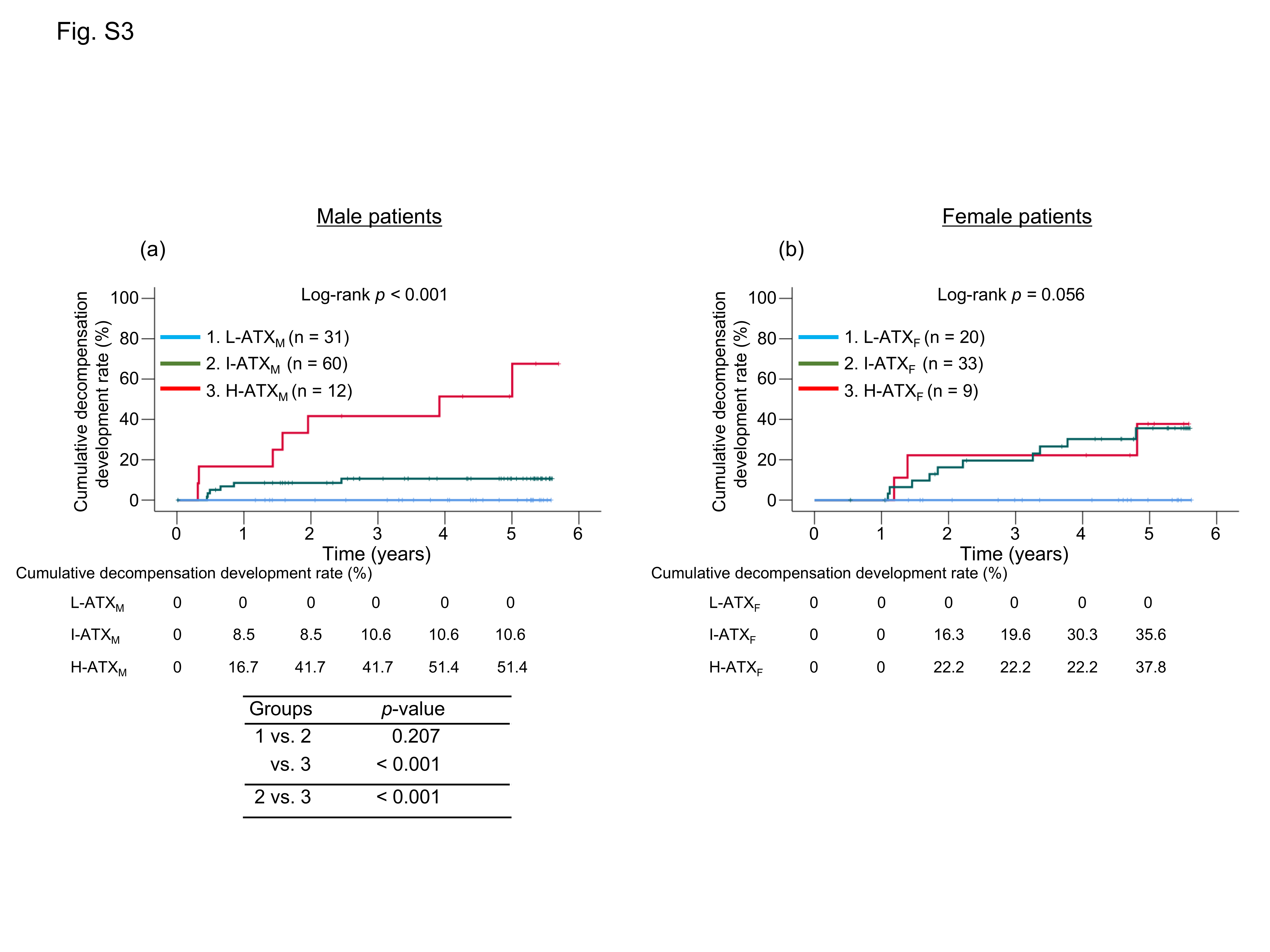

Supplement: S3 Fig — (a) Male patients; (b) female patients. L-ATXmen (M)/women (W), low-ATX group; I-ATXM/W, intermediate-ATX group; H-ATXM/W, high-ATX group. (TIF) [file pone.0347310.s003.tif]
