# Supplementary material for: Clinical usefulness of serum autotaxin levels for predicting decompensation development and prognosis in patients with compensated cirrhosis
Source: PLoS One. 2026 Apr 9;21(4):e0347310. doi: 10.1371/journal.pone.0347310 (PMC13065023; doi:10.1371/journal.pone.0347310)
Supplement: S1 Table — (DOCX) [file pone.0347310.s004.docx]

**S1 Table. Clinical characteristics by gender**

| Variable | Men | Women | *p* value |
| --- | --- | --- | --- |
| Patients, n (%) | 130 (61.9) | 80 (38.1) |  |
| Age (years) | 68.0 (58.0–76.3) | 72.0 (58.5–79.8) | 0.243 |
| Etiology |  |  |  |
| HBV/HCV/Alcohol/other, n | 15/24/67/24 | 6/17/11/46 | < 0.001 |
| Decompensated cirrhosis, n (%) | 27 (20.8) | 18 (22.5) | 0.767 |
| Child-Pugh score | 5 (5–6) | 5 (5–7) | 0.632 |
| MELD score | 8 (7–10) | 7 (6–9) | 0.044 |
| ALBI score | −2.65 (−2.90–−2.22) | −2.55 (−2.87–−2.06) | 0.303 |
| Total bilirubin (mg/dL) | 0.9 (0.7–1.2) | 0.7 (0.6–1.1) | 0.055 |
| Albumin (g/dL) | 4.0 (3.6–4.2) | 3.9 (3.3–4.2) | 0.079 |
| Prothrombin time INR | 1.07 (1.00–1.19) | 1.06 (1.00–1.19) | 0.863 |
| Creatinine (mg/dL) | 0.9 (0.8–1.2) | 0.7 (0.6–0.9) | < 0.001 |
| Sodium (mEq/L) | 140 (138–142) | 140 (139–142) | 0.359 |
| Platelet (x10^4^/µl) | 11.8 (8.6–16.1) | 13.2 (9.6–17.5) | 0.124 |
| Autotaxin (mg/L) | 1.189 (0.957–1.607) | 1.560 (1.282–1.890) | < 0.001 |

Continuous variables are shown as median (interquartile range). Statistical analysis was performed using the chi-squared test or the Mann-Whitney U test, as appropriate. ALBI, albumin-bilirubin; HBV, hepatitis B virus; HCV, hepatitis C virus; INR, international normalized ratio; MELD, model for end-stage liver disease.
