# Supplementary material for: Clinical usefulness of serum autotaxin levels for predicting decompensation development and prognosis in patients with compensated cirrhosis
Source: PLoS One. 2026 Apr 9;21(4):e0347310. doi: 10.1371/journal.pone.0347310 (PMC13065023; doi:10.1371/journal.pone.0347310)
Supplement: S2 Table — (DOCX) [file pone.0347310.s005.docx]

**S2 Table. Correlation between serum autotaxin levels and baseline characteristics**

| Variable | Correlation coefficient | | *p* value |
| --- | --- | --- | --- |
| Age (years) | 0.073 | 0.291 | |
| Child-Pugh score | 0.594 | < 0.001 | |
| MELD score | 0.417 | < 0.001 | |
| ALBI score | 0.625 | < 0.001 | |
| Total bilirubin (mg/dL) | 0.402 | < 0.001 | |
| Albumin (g/dL) | −0.611 | < 0.001 | |
| Prothrombin time INR | 0.525 | < 0.001 | |
| Creatinine (mg/dL) | −0.055 | 0.427 | |
| Sodium (mEq/L) | −0.349 | < 0.001 | |
| Platelet (x10^4^/µl) | −0.388 | < 0.001 | |

ALBI, albumin-bilirubin; INR, international normalized ratio; MELD, model for end-stage liver disease.
