# Supplementary material for: Clinical usefulness of serum autotaxin levels for predicting decompensation development and prognosis in patients with compensated cirrhosis
Source: PLoS One. 2026 Apr 9;21(4):e0347310. doi: 10.1371/journal.pone.0347310 (PMC13065023; doi:10.1371/journal.pone.0347310)
Supplement: S3 Table — (DOCX) [file pone.0347310.s006.docx]

**S3 Table. Univariate analysis of factors associated with mortality in all patients and according to sex**

**All patients**

| Variable | HR (95% CI) | *p* value |
| --- | --- | --- |
| Gender (Women) | 1.155 (0.630–2.118) | 0.640 |
| Age (years) | 1.010 (0.985–1.036) | 0.430 |
| Decompensated cirrhosis | 22.427 (10.810–46.527) | < 0.001 |
| Child-Pugh score | 2.279 (1.951–2.662) | < 0.001 |
| MELD score | 1.233 (1.180–1.288) | < 0.001 |
| ALBI score | 9.201 (6.029–14.044) | < 0.001 |
| Total bilirubin (mg/dL) | 1.596 (1.424–1.788) | < 0.001 |
| Albumin (g/dL) | 0.093 (0.057–0.152) | < 0.001 |
| Prothrombin time INR | 5.446 (3.479–8.526) | < 0.001 |
| Creatinine (mg/dL) | 2.316 (1.233–4.351) | 0.009 |
| Sodium (mEq/L) | 0.694 (0.623–0.772) | < 0.001 |
| Platelet (x10^4^/µl) | 0.874 (0.816–0.935) | < 0.001 |
| Autotaxin (mg/L) | 4.721 (3.361–6.631) | < 0.001 |

**Male patients**

| Variable | HR (95% CI) | *p* value |
| --- | --- | --- |
| Age (years) | 1.010 (0.976–1.046) | 0.567 |
| Decompensated cirrhosis | 26.507 (9.688–72.528) | < 0.001 |
| Child-Pugh score | 2.105 (1.742–2.543) | < 0.001 |
| MELD score | 1.203 (1.140–1.269) | < 0.001 |
| ALBI score | 6.908 (4.200–11.361) | < 0.001 |
| Total bilirubin (mg/dL) | 1.481 (1.288–1.702) | < 0.001 |
| Albumin (g/dL) | 0.118 (0.067–0.209) | < 0.001 |
| Prothrombin time INR | 4.228 (2.425–7.373) | < 0.001 |
| Creatinine (mg/dL) | 2.714 (1.223–6.025) | 0.014 |
| Sodium (mEq/L) | 0.729 (0.634–0.839) | < 0.001 |
| Platelet (x10^4^/µl) | 0.887 (0.810–0.971) | 0.009 |
| Autotaxin (mg/L) | 6.781 (3.976–11.564) | < 0.001 |

**Female patients**

| Variable | HR (95% CI) | *p* value |
| --- | --- | --- |
| Age (years) | 1.008 (0.973–1.045) | 0.649 |
| Decompensated cirrhosis | 19.698 (6.314–61.453) | < 0.001 |
| Child-Pugh score | 2.715 (1.990–3.705) | < 0.001 |
| MELD score | 1.433 (1.276–1.609) | < 0.001 |
| ALBI score | 18.713 (7.508–46.638) | < 0.001 |
| Total bilirubin (mg/dL) | 3.172 (2.137–4.708) | < 0.001 |
| Albumin (g/dL) | 0.056 (0.021–0.151) | < 0.001 |
| Prothrombin time INR | 206.186 (30.313–1402.460) | < 0.001 |
| Creatinine (mg/dL) | 2.260 (0.838–6.091) | 0.107 |
| Sodium (mEq/L) | 0.591 (0.481–0.726) | < 0.001 |
| Platelet (x10^4^/µl) | 0.850 (0.767–0.942) | 0.002 |
| Autotaxin (mg/L) | 3.919 (2.325–6.605) | < 0.001 |

ALBI, albumin-bilirubin; CI, confidence interval; HR, hazard ratio; INR, international normalized ratio; MELD, model for end-stage liver disease.
