# Supplementary material for: Clinical usefulness of serum autotaxin levels for predicting decompensation development and prognosis in patients with compensated cirrhosis
Source: PLoS One. 2026 Apr 9;21(4):e0347310. doi: 10.1371/journal.pone.0347310 (PMC13065023; doi:10.1371/journal.pone.0347310)
Supplement: S4 Table — (DOCX) [file pone.0347310.s007.docx]

**S4 Table. Significant factors associated with mortality according to sex**

**Male patients**

|  | Univariate | |  | Multivariate | |
| --- | --- | --- | --- | --- | --- |
| Variable | HR (95%CI) | *p* value |  | HR (95%CI) | *p* value |
| Age (years) | 1.010 (0.976–1.046) | 0.567 |  |  |  |
| Decompensated cirrhosis | 26.507 (9.688–72.528) | < 0.001 |  | 11.516 (3.302–40.160) | < 0.001 |
| Child-Pugh score | 2.105 (1.742–2.543) | < 0.001 |  |  |  |
| MELD score | 1.203 (1.140–1.269) | < 0.001 |  |  |  |
| ALBI score | 6.908 (4.200–11.361) | < 0.001 |  |  |  |
| Sodium (mEq/L) | 0.729 (0.634–0.839) | < 0.001 |  | 0.735 (0.621–0.869) | < 0.001 |
| Platelet (x10^4^/µl) | 0.887 (0.810–0.971) | 0.009 |  |  |  |
| Autotaxin (mg/L) | 6.781 (3.976–11.564) | < 0.001 |  | 2.788 (1.394–5.574) | 0.004 |

**Female patients**

|  | Univariate | |  | Multivariate | |
| --- | --- | --- | --- | --- | --- |
| Variable | HR (95%CI) | *p* value |  | HR (95%CI) | *p* value |
| Age (years) | 1.008 (0.973–1.045) | 0.649 |  |  |  |
| Decompensated cirrhosis | 19.698 (6.314–61.453) | < 0.001 |  |  |  |
| Child-Pugh score | 2.715 (1.990–3.705) | < 0.001 |  | 2.715 (1.990–3.705) | < 0.001 |
| MELD score | 1.433 (1.276–1.609) | < 0.001 |  |  |  |
| ALBI score | 18.713 (7.508–46.638) | < 0.001 |  |  |  |
| Sodium (mEq/L) | 0.591 (0.481–0.726) | < 0.001 |  |  |  |
| Platelet (x10^4^/µl) | 0.850 (0.767–0.942) | 0.002 |  |  |  |
| Autotaxin (mg/L) | 3.919 (2.325–6.605) | < 0.001 |  |  |  |

ALBI, albumin-bilirubin; CI, confidence interval; HR, hazard ratio; MELD, model for end-stage liver disease.
