# Supplementary material for: Clinical usefulness of serum autotaxin levels for predicting decompensation development and prognosis in patients with compensated cirrhosis
Source: PLoS One. 2026 Apr 9;21(4):e0347310. doi: 10.1371/journal.pone.0347310 (PMC13065023; doi:10.1371/journal.pone.0347310)
Supplement: S5 Table — (DOCX) [file pone.0347310.s008.docx]

**S5 Table. Univariate analysis of factors associated with mortality in patients with compensated and decompensated cirrhosis**

**Compensated cirrhosis**

| Variable | HR (95% CI) | *p* value |
| --- | --- | --- |
| Gender (Women) | 1.391 (0.424–4.561) | 0.586 |
| Age (years) | 1.030 (0.976–1.086) | 0.283 |
| Child-Pugh score | 3.391 (1.615–7.119) | 0.001 |
| MELD score | 1.243 (0.985–1.568) | 0.067 |
| ALBI score | 11.572 (2.920–45.859) | < 0.001 |
| Total bilirubin (mg/dL) | 6.831 (1.696–27.507) | 0.007 |
| Albumin (g/dL) | 0.152 (0.046–0.498) | 0.002 |
| Prothrombin time INR | 270.444 (2.224–32893.641) | 0.022 |
| Creatinine (mg/dL) | 0.985 (0.153–6.338) | 0.987 |
| Sodium (mEq/L) | 0.775 (0.620–0.969) | 0.025 |
| Platelet (x10^4^/µl) | 0.905 (0.799–1.025) | 0.115 |
| Autotaxin (mg/L) | 10.862 (4.022–29.334) | < 0.001 |

**Decompensated cirrhosis**

| Variable | HR (95% CI) | *p* value |
| --- | --- | --- |
| Gender (Women) | 0.958 (0.472–1.942) | 0.905 |
| Age (years) | 0.997 (0.965–1.030) | 0.852 |
| Child-Pugh score | 1.692 (1.315–2.177) | < 0.001 |
| MELD score | 1.108 (1.047–1.172) | < 0.001 |
| ALBI score | 3.489 (1.917–6.351) | < 0.001 |
| Total bilirubin (mg/dL) | 1.282 (1.136–1.447) | < 0.001 |
| Albumin (g/dL) | 0.242 (0.118–0.494) | < 0.001 |
| Prothrombin time INR | 2.007 (1.091–3.694) | 0.025 |
| Creatinine (mg/dL) | 1.525 (0.821–2.832) | 0.181 |
| Sodium (mEq/L) | 0.727 (0.632–0.838) | < 0.001 |
| Platelet (x10^4^/µl) | 0.995 (0.923–1.073) | 0.906 |
| Autotaxin (mg/L) | 1.639 (1.047–2.566) | 0.031 |

ALBI, albumin-bilirubin; CI, confidence interval; HR, hazard ratio; INR, international normalized ratio; MELD, model for end-stage liver disease.
