# Supplementary material for: Clinical usefulness of serum autotaxin levels for predicting decompensation development and prognosis in patients with compensated cirrhosis
Source: PLoS One. 2026 Apr 9;21(4):e0347310. doi: 10.1371/journal.pone.0347310 (PMC13065023; doi:10.1371/journal.pone.0347310)
Supplement: S6 Table — (DOCX) [file pone.0347310.s009.docx]

**S6 Table. Significant factors associated with mortality in patients with decompensated cirrhosis**

|  | Univariate | |  | Multivariate | |
| --- | --- | --- | --- | --- | --- |
| Variable | HR (95%CI) | *p* value |  | HR (95%CI) | *p* value |
| Gender (Women) | 0.958 (0.472–1.942) | 0.905 |  |  |  |
| Age (years) | 0.997 (0.965–1.030) | 0.852 |  |  |  |
| Child-Pugh score | 1.692 (1.315–2.177) | < 0.001 |  |  |  |
| MELD score | 1.108 (1.047–1.172) | < 0.001 |  |  |  |
| ALBI score | 3.489 (1.917–6.351) | < 0.001 |  | 2.895 (1.583–5.295) | < 0.001 |
| Sodium (mEq/L) | 0.727 (0.632–0.838) | < 0.001 |  | 0.736 (0.630–0.861) | < 0.001 |
| Autotaxin (mg/L) | 1.639 (1.047–2.566) | 0.031 |  |  |  |

ALBI, albumin-bilirubin; CI, confidence interval; HR, hazard ratio; MELD, model for end-stage liver disease.
