# Supplementary material for: Clinical usefulness of serum autotaxin levels for predicting decompensation development and prognosis in patients with compensated cirrhosis
Source: PLoS One. 2026 Apr 9;21(4):e0347310. doi: 10.1371/journal.pone.0347310 (PMC13065023; doi:10.1371/journal.pone.0347310)
Supplement: S7 Table — (DOCX) [file pone.0347310.s010.docx]

**S7 Table. Univariate analysis of factors associated with decompensation development in all patients and according to sex**

**All patients**

| Variable | HR (95% CI) | *p* value |
| --- | --- | --- |
| Gender (Women) | 1.650 (0.765–3.561) | 0.202 |
| Age (years) | 1.027 (0.994–1.062) | 0.114 |
| Child-Pugh score | 3.791 (2.307–6.228) | < 0.001 |
| MELD score | 1.165 (0.996–1.363) | 0.056 |
| ALBI score | 10.862 (4.386–26.903) | < 0.001 |
| Total bilirubin (mg/dL) | 2.380 (0.877–6.464) | 0.089 |
| Albumin (g/dL) | 0.134 (0.062–0.291) | < 0.001 |
| Prothrombin time INR | 266.842 (9.381–5485.454) | < 0.001 |
| Creatinine (mg/dL) | 0.574 (0.151–2.182) | 0.415 |
| Sodium (mEq/L) | 0.891 (0.765–1.038) | 0.137 |
| Platelet (x10^4^/µl) | 0.889 (0.817–0.967) | 0.006 |
| Autotaxin (mg/L) | 6.136 (3.399–11.074) | < 0.001 |

**Male patients**

| Variable | HR (95% CI) | *p* value |
| --- | --- | --- |
| Age (years) | 1.007 (0.962–1.055) | 0.764 |
| Child-Pugh score | 3.453 (1.572–7.587) | 0.002 |
| MELD score | 1.065 (0.835–1.359) | 0.611 |
| ALBI score | 11.546 (3.156–42.242) | < 0.001 |
| Total bilirubin (mg/dL) | 1.763 (0.403–7.721) | 0.452 |
| Albumin (g/dL) | 0.088 (0.023–0.328) | < 0.001 |
| Prothrombin time INR | 42.966 (0.300–6159.523) | 0.138 |
| Creatinine (mg/dL) | 0.537 (0.069–4.149) | 0.551 |
| Sodium (mEq/L) | 0.915 (0.747–1.121) | 0.391 |
| Platelet (x10^4^/µl) | 0.846 (0.733–0.975) | 0.021 |
| Autotaxin (mg/L) | 7.348 (3.300–16.359) | < 0.001 |

**Female patients**

| Variable | HR (95% CI) | *p* value |
| --- | --- | --- |
| Age (years) | 1.046 (0.991–1.103) | 0.103 |
| Child-Pugh score | 4.086 (2.112–7.903) | < 0.001 |
| MELD score | 1.353 (1.112–1.647) | 0.003 |
| ALBI score | 9.425 (2.662–33.364) | < 0.001 |
| Total bilirubin (mg/dL) | 5.140 (1.461–18.087) | 0.011 |
| Albumin (g/dL) | 0.174 (0.060–0.499) | 0.001 |
| Prothrombin time INR | 37367.371 (78.497–18046171.40) | < 0.001 |
| Creatinine (mg/dL) | 1.228 (0.205–7.376) | 0.822 |
| Sodium (mEq/L) | 0.786 (0.595–1.039) | 0.091 |
| Platelet (x10^4^/µl) | 0.905 (0.820–0.999) | 0.047 |
| Autotaxin (mg/L) | 5.661 (2.083–15.383) | < 0.001 |

ALBI, albumin-bilirubin; CI, confidence interval; HR, hazard ratio; INR, international normalized ratio; MELD, model for end-stage liver disease.
