# Supplementary material for: Clinical usefulness of serum autotaxin levels for predicting decompensation development and prognosis in patients with compensated cirrhosis
Source: PLoS One. 2026 Apr 9;21(4):e0347310. doi: 10.1371/journal.pone.0347310 (PMC13065023; doi:10.1371/journal.pone.0347310)
Supplement: S8 Table — (DOCX) [file pone.0347310.s011.docx]

**S8 Table. Significant factors associated with decompensation development according to sex**

**Male patients**

|  | Univariate | |  | Multivariate | |
| --- | --- | --- | --- | --- | --- |
| Variable | HR (95%CI) | *p* value |  | HR (95%CI) | *p* value |
| Age (years) | 1.007 (0.962–1.055) | 0.764 |  |  |  |
| Child-Pugh score | 3.453 (1.572–7.587) | 0.002 |  |  |  |
| ALBI score | 11.546 (3.156–42.242) | < 0.001 |  |  |  |
| Platelet (x10^4^/µl) | 0.846 (0.733–0.975) | 0.021 |  |  |  |
| Autotaxin (mg/L) | 7.348 (3.300–16.359) | < 0.001 |  | 7.348 (3.300–16.359) | < 0.001 |

**Female patients**

|  | Univariate | |  | Multivariate | |
| --- | --- | --- | --- | --- | --- |
| Variable | HR (95%CI) | *p* value |  | HR (95%CI) | *p* value |
| Age (years) | 1.046 (0.991–1.103) | 0.103 |  | 1.097 (1.021–1.178) | 0.011 |
| Child-Pugh score | 4.086 (2.112–7.903) | < 0.001 |  | 5.529 (2.178–14.037) | < 0.001 |
| MELD score | 1.353 (1.112–1.647) | 0.003 |  |  |  |
| ALBI score | 9.425 (2.662–33.364) | < 0.001 |  |  |  |
| Sodium (mEq/L) | 0.786 (0.595–1.039) | 0.091 |  |  |  |
| Platelet (x10^4^/µl) | 0.905 (0.820–0.999) | 0.047 |  | 0.840 (0.729–0.967) | 0.015 |
| Autotaxin (mg/L) | 5.661 (2.083–15.383) | < 0.001 |  | 4.046 (1.248–13.114) | 0.020 |

ALBI, albumin-bilirubin; CI, confidence interval; HR, hazard ratio; MELD, model for end-stage liver disease.
