# Supplementary material for: Clinical usefulness of serum autotaxin levels for predicting decompensation development and prognosis in patients with compensated cirrhosis
Source: PLoS One. 2026 Apr 9;21(4):e0347310. doi: 10.1371/journal.pone.0347310 (PMC13065023; doi:10.1371/journal.pone.0347310)
Supplement: Attachment — Submitted filename: Response to Reviewers R2.docx [file pone.0347310.s014.docx]

**RESPONSES TO THE REVIEWER**

We appreciate the opportunity to submit a revised version of our manuscript entitled “**Clinical usefulness of serum autotaxin** levels for predicting decompensation development and prognosis in patients with compensated cirrhosis” to *PLOS One*. We also appreciate the reviewers' constructive comments and suggestions. Our point-by-point responses to the reviewers’ comments are listed below.

Reviewer Comments:

**Reviewer #1**

The authors addressed most of the reviewers' concerns. However, there is disagreement regarding the ATX cutoff values. While the text states that "all analyses were stratified by sex using sex-
specific cutoff values. Patients were categorized into three groups according to serum ATX levels," Table 1 categorizes all case data into three groups instead of separating by sex. Therefore, for example, it is unclear whether a female case with an ATX of 1.8 is classified as Intermediate or High. The data should be standardized to sex-specific cutoff values as stated in the text. Furthermore, the authors should discuss why ATX levels are higher in female than in male.

**Responses:** We apologize that the description of the patient classification in the Methods section may have been misleading. First, we established cutoff values based on the overall cohort and performed stratified analyses. Subsequently, given that sex differences in ATX levels have been reported, we also performed sex-stratified analyses using sex-specific cutoff values. We believe that this stepwise approach, from analyses of the overall cohort to sex-stratified analyses, is reasonable and methodologically appropriate. We have revised the description of the patient classification in the Methods section (lines 134–137 in the revised manuscript).

In this study, serum ATX levels were significantly higher in female patients than in male patients, in line with previous reports [Refs. 18, 25]. Although the mechanisms underlying this sex difference remain unclear, previous studies have suggested that ATX expression may be influenced by sex steroids, including estrogen (Refs. 18, 29). Thus, this difference may need to be considered when using ATX as a biomarker for decompensation and prognosis. We have added these descriptions to the Discussion section (lines 398–394 in the revised manuscript).

**Reviewer #2**

The authors have adequately addressed the issues I raised, and the manuscript has been substantially improved. In its current form, I consider it acceptable for publication.

**Responses:** We are grateful for the reviewer’s encouraging comments.
